# The first occurrence of an avian-style respiratory infection in a non-avian dinosaur

**DOI:** 10.1038/s41598-022-05761-3

**Published:** 2022-02-10

**Authors:** D. Cary Woodruff, Ewan D. S. Wolff, Mathew J. Wedel, Sophie Dennison, Lawrence M. Witmer

**Affiliations:** 1 Great Plains Dinosaur Museum, Malta, MT USA; 2 Royal Ontario Museum, University of Toronto, Toronto, ON Canada; 3 Department of Ecology and Evolutionary Biology, University of Toronto, Toronto, ON Canada; 4 Museum of the Rockies, Bozeman, MT USA; 5 University of New Mexico Honors College, Albuquerque, NM USA; 6 Nevada Science Center, Henderson, NV USA; 7 College of Osteopathic Medicine of the Pacific, Western University of Health Sciences, Pomona, CA USA; 8 TeleVet Imaging Solutions, PLLC, Oakton, VA USA; 9 Biomedical Sciences, Ohio University Heritage College of Osteopathic Medicine, Athens, OH USA

**Keywords:** Evolution, Zoology, Diseases

## Abstract

Other than repaired fractures, osteoarthritis, and periosteal reaction, the vertebrate fossil record has limited evidence of non-osseous diseases. This difficulty in paleontological diagnoses stems from (1) the inability to conduct medical testing, (2) soft-tissue pathologic structures are less likely to be preserved, and (3) many osseous lesions are not diagnostically specific. However, here reported for the first time is an avian-style respiratory disorder in a non-avian dinosaur. This sauropod presents irregular bony pathologic structures stemming from the pneumatic features in the cervical vertebrae. As sauropods show well-understood osteological correlates indicating that respiratory tissues were incorporated into the post-cranial skeleton, and thus likely had an ‘avian-style’ form of respiration, it is most parsimonious to identify these pathologic structures as stemming from a respiratory infection. Although several extant avian infections produce comparable symptoms, the most parsimonious is airsacculitis with associated osteomyelitis. From actinobacterial to fungal in origin, airsacculitis is an extremely prevalent respiratory disorder in birds today. While we cannot pinpoint the specific infectious agent that caused the airsacculitis, this diagnosis establishes the first fossil record of this disease. Additionally, it allows us increased insight into the medical disorders of dinosaurs from a phylogenetic perspective and understanding what maladies plagued the “fearfully great lizards”.

## Introduction

Our understanding of the fossil record is predominantly represented by permineralized osseous tissues (bones and teeth); therefore, our basic foundations for understanding extinct animals are directly built upon their own biomineralized scaffold. The historical adage was that fossilization could only occur with osseous tissues; consequently, unless preserved on or within a skeletal element, we could not derive any information with certainty. Other biological aspects, including anatomy, physiology, metabolism, behavior, and coloration, not derived from bone were drawn based on comparisons to living relatives. However, within the past several decades, our understanding of the process of fossilization is undergoing a paradigm shift. Within dinosaur paleontology, not only are myological, connective, and cutaneous tissues being reported with increasing frequency^1–6^, but so too are melanin containing organelles^7,8^. Likewise, dinosaurian osteology itself is helping to reveal the nature of some overlying tissues—nostril size and position^9^, ‘cheeks’^10–12^, facial sensory^13^, nasal airflow^14^, and cranial vascular anatomy^15^. These new findings are literally helping to more accurately ‘flesh out’ dinosaurs and ‘brighten’ our views and reconstructions of them^10^.

While these new findings and research are leading the forefront of paleontological research, finer life history details still represent intimate and exceedingly rare glimpses of an animal’s life. Trackways have not only demonstrated herding behaviors^16^, but also critical moments in predator–prey interactions^17,18^, and even courtship displays^19^. Nests and eggs have revealed such details as parental care^20^, incubation practices^21^, and egg care^22^. Even the bones themselves have provided details of an individual’s life—more so than metabolic rate or life history, bone pathology has revealed breaks and healing^23^, compensation growth^24^, infectious and non-infectious arthritis^25,26^, and even infectious disease^27,28^. These finer details help us to paint a picture of the animal and understand its life.

When and how diseases evolved plays a tremendous role in better understanding and fighting them^29^. Previously, some osteological indicators of disease^27^ and infections were misinterpreted as evidence of combat. Such recasting of pathology may pinpoint causation, potential etiologic agents responsible^27^, and in turn trace the evolutionary history of disease.

In terms of non-avian dinosaurs, our record of paleopathology is limited. What we do know is that disorders such as trauma^23,30,31^, various forms of arthritis (such as spondyloarthropathy;^32^, neoplasia^32–34^, osteomyelitis^32^, vascular parasites^35^, and even transmissible diseases (such as trichomonosis^27^) were present.

Today, respiratory diseases of archosaurs include inflammatory, infectious (bacterial, viral, fungal, mycobacterial, parasitic), and neoplastic etiologies^36,37^. A mycobacterial-like infection in a 245-million-year-old marine reptile^38^ is the oldest described. This specimen exhibits parosteal proliferation on dorsal ribs with histology that reveals tissue changes similar to modern cases of mycobacterial pulmonary infection. As mycobacterial infections are prevalent in numerous varieties today, understanding their origins and evolutionary history is of considerable interest. Beyond what can be extrapolated from the extant phylogenetic bracket of Archosauria^10,27^, there is no guidance from prior research regarding dinosaurian pulmonary disease derived from either Crocodilia or Aves. In this paper the authors report on the first known case of an avian-style pulmonary infectious disease in a non-avian dinosaur.

## Materials and methods

### Specimen

MOR 7029—an immature individual tentatively identified here as an indeterminate diplodocine—was originally collected in 1990 from the undivided Morrison Formation of southwest Montana. The remains originally collected consist of a complete skull with the first seven associated and articulated cervical vertebrae. Recovered from the Lower O’Hair Quarry (MOR locality no M-048), this locality—subsequently re-opened from 2013 to 2015—has produced additional postcranial material from this individual. In addition to MOR 7029, this locality has produced additional, largely complete diplodocid specimens, infantile *Camarasaurus* cranial remains^39^, along with the first *Hesperosaurus mjosi*^40^, the first *Camptosaurus*^40^, the first *Allosaurus*, and the first sphenodontid from Montana.

### Examination techniques

Cervical vertebrae from MOR 7029 exhibiting macroscopic bone lesions at the site of air sac insertion were examined and photographed firsthand by DCW and EDSW. Two cervical vertebrae—cervicals (Cv) 6 and 7—were scanned via computed tomography (CT scan) conducted by the Phillips County Hospital in Malta, Montana, USA using a GE Revolution CT scanner (120 KVP, 149 mA, 50 cm scan field of view, 1.25 mm slice thickness, bone reconstruction algorithm), and at Advanced Medical Imaging at Bozeman Deaconess Hospital in Bozeman, Montana, USA using a Toshiba Aquilion 64 CT Scanner. Scan DICOM data was uploaded into the DICOM viewers Novarad and OsiriX for multiplanar assessment, with individual planes being analyzed using the image processing program ImageJ^41^.

### Institutional abbreviations

AMNH, American Museum of Natural History, New York, NY, USA; MOR, Museum of the Rockies, Bozeman, MT, USA; OMNH, Sam Noble Museum, Norman, OK, USA; SMA, Sauriermuseum Aathal, Aathal, Switzerland; USNM, United States National Museum, Washington, D.C., USA.

## Description

### Macroscopic examination

**Figure 1:**
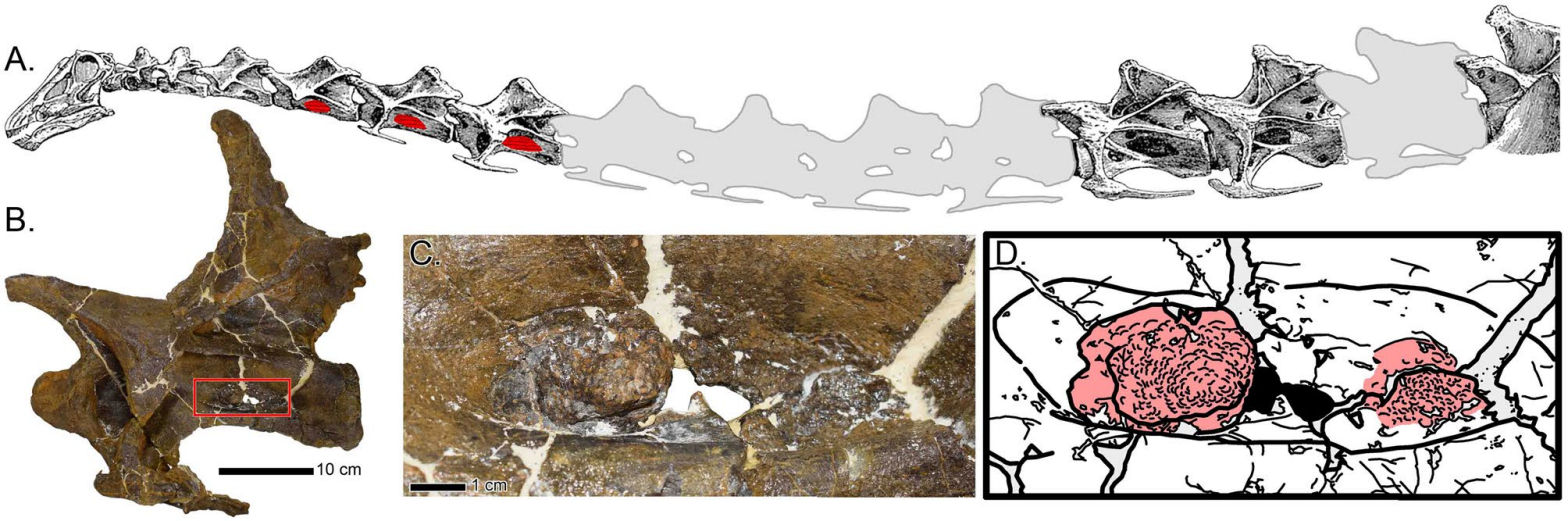
Pathologic pneumatic tissue in MOR 7029. (**A**) Schematic map of the neck of *Diplodocus* (Ref.^104^; bones not present in grey), with the pathologic structures denoted in red. (**B**) Cervical 5 of MOR 7029 with red box highlighting the pathologic structure; close up in (**C**) with interpretative drawing in (**D**) (by DCW) (pathology in red).

While sauropodomorph vertebral pathology is not uncommon^30,42–50^, the features in MOR 7029 cervical vertebrae (Cv) 5–7 can immediately be recognized as a novel type of pathology (Fig. 1). Sauropod cervical vertebrae are characterized by elaborate laminae and complex pneumatic architecture. The cervical vertebrae of MOR 7029 display evident taphonomic distortion (including shearing and crushing), but there are abnormal structures discussed herein that do not occur within displaced or misshaped bone. In these vertebrae, the asymmetry, morphology, location and texture changes of these structures serve to highlight a pathologic origin associated with the pneumatic fossae margins. Though highly variable, these structures have a superficial multifocal nodular appearance causing a very undulating and non-uniform surface. The bony proliferations vary in size and extent. The largest discrete area of proliferation is identified within the left pneumatic fossa of Cv5 and measures 2.8 cm craniocaudally by 2.1 cm dorsoventrally—approximately 23% of the pneumatic fossa’s length. The height of the proliferations of bone protruding from the pneumatic fossae is equally variable; between Cv5 and Cv7, they vary 0.5 to 1 cm in relief from the bone surface and occur on both sides.

### CT assessment

CT scans were conducted to examine internal and microstructural anatomy. Sauropod vertebrae are known for the high degree of pneumatization that develops in increasing complexity through evolution^51^ and ontogeny^52^. Within sauropod cervical vertebrae, there are several forms of pneumatic architecture. Adjacent to the laterally oriented pneumatic fossae (a.k.a and inaccurately referred to as “pleurocoels”, sensu^51^) are either camerae or camellae^51^. The difference between camerae and camellae is their shape, size, and branching pattern: camera—round cavity, 5–150 mm in size, septal thickness of 2–10 mm, and a regular branching pattern; camella—angular cavity, 2–20 mm in size, septal thickness of 1–3 mm, and an irregular branching pattern^53^. Within the condyle and cotyle, one can see either symmetrically distributed (laterally and circumferentially along the face) camerae or camellae. Typically, within the neural arch, several fossae, camerae, and camellae can be observed in proximity to the neural canal, diapophyses, zygapophyses, and neural spine. All of these pneumatic structures can vary in form, size, and location because of phylogeny^51^ and ontogeny^52^. While pervasive internal pneumatization occurs throughout the vertebra, the medial septa are generally apneumatic, but infrequent camellae can be observed^51,52^.

On CT images of MOR 7029’s Cv6 and 7, there are proliferative superficial lesions that protrude from the surfaces of the pneumatic fossae on the vertebrae into the fossae suggestive of periosteal reaction (Fig. 1). These proliferations are irregular and non-uniform with both discrete (Cv6) and more extensive (ventral Cv7) distributions limited to and seen along the pneumatic fossal surface. The periosteal proliferative changes are present bilaterally but are asymmetrical.

**Figure 2:**
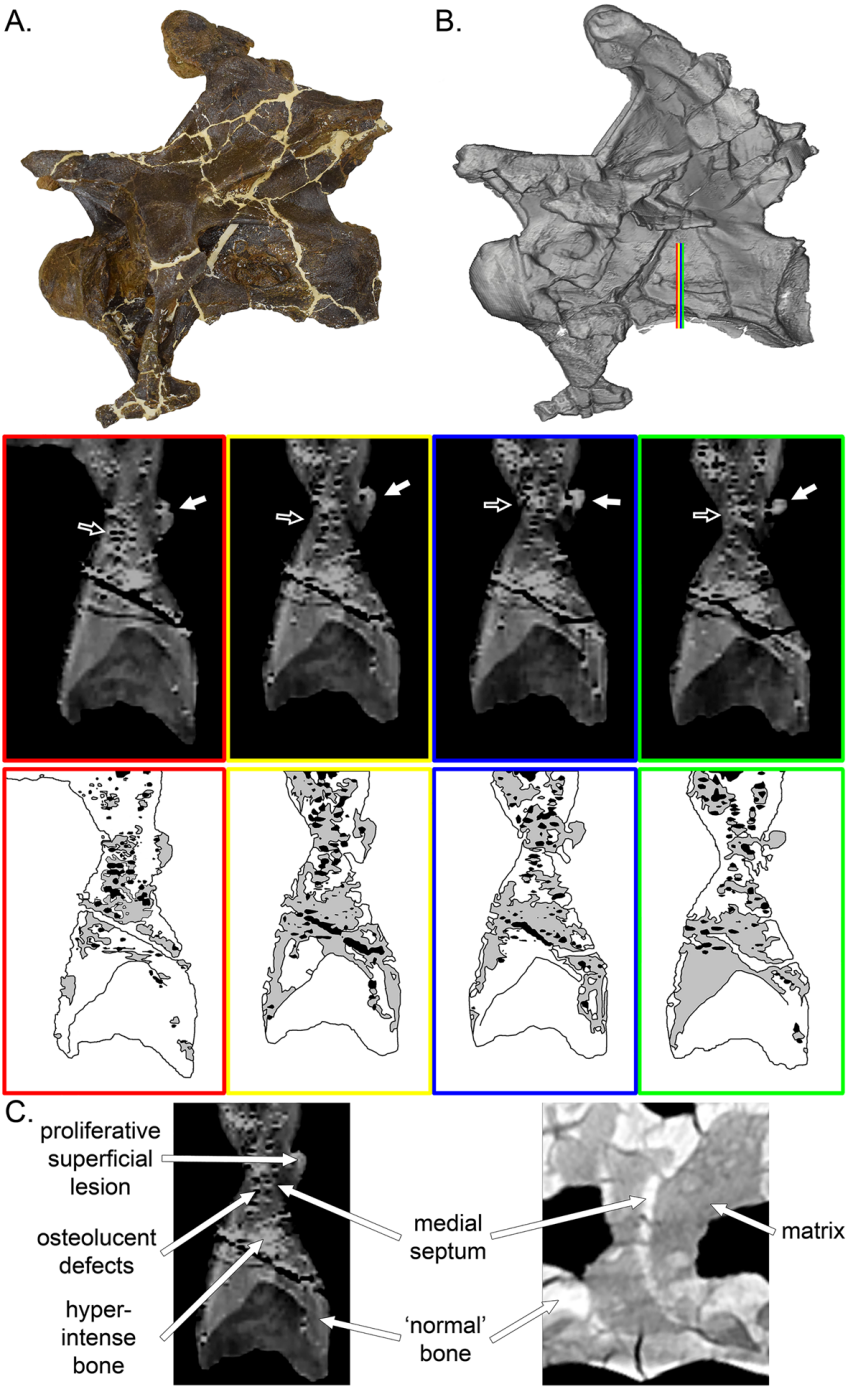
CT scans of cervical 7 of MOR 7029. Photograph and scan model of the vertebra ((**A,B**) respectively). The colored lines in (**B**) correspond to the scan slices (and scan interpretative drawings below). White arrows point to the external feature, while black arrows denote the hyperintense bone and irregular voids. (**C**) Comparison of the abnormal tissue composition of MOR 7029 (left), compared to that of a ‘normal’ diplodocine (right). Text and white arrows indicate the various features different shared/differentiated between the two. For the interpretative drawings, white = ‘normal’ bone, grey = hyperintense bone, black = irregular voids.

In addition to the superficial proliferative periosteal changes observed, intraosseous defects are also noted on multiplanar reconstructions of the vertebrae (Fig. 2) There are multiple, small osteolucent defects identified that are disorganized and variable in appearance, both ovoid and more tubular to angular in shape, and arranged in a haphazard manner with variable wall thickness noted. Bone adjacent to these lesions is notably and non-uniformly hyperattenuating. In several areas in all three vertebrae beam hardening artifact is associated with these very hyperattenuating regions which would indicate the likely presence of metal deposits. The osteolucent defects are distributed multifocally and non-uniformly throughout the cotyle, condyle and median septum, with the greatest density of distribution noted surrounding the abnormal pneumatic fossae.

The presence of a large number of osteolucent structures within the median septum combined with the generally haphazard arrangement of the structures with non-uniform walls is abnormal as the median septum is considered apneumatic. The haphazard arrangement is inconsistent with the expected pattern of pneumatic structures even considering individual variation. This may suggest chronic disruption of normal pneumatic structures such as occurs in bone with chronic pathology in other species, or reflect multifocal foci of chronic bone lysis without re-ossification. The periosteal proliferation is limited to being within the pneumatic fossae and as such is strongly suggestive of reaction to pathology of the adjacent air sacs.

## Discussion

In light of these strikingly evident bone lesions, the immediate question is what was their causation? In veterinary necropsies, histological sampling, immunohistochemistry, cultures, enzyme-linked immunosorbent assay (ELISA), and polymerase chain reaction (PCR) tests can be used to assess and determine the etiopathogenesis of bone pathology. Unfortunately, in the paleontological record, causation is much more problematic to determine. As we are largely left with permineralized bone, our preserved record may be biased in one way or another toward more or less developed lesions.

The location of bone lesions may aid significantly in differential diagnosis. If the lesions of MOR 7029 were associated with healed fractured bone, we could refer to them as a fracture callus (which are reported in Morrison Formation sauropods [ex.^54^]). Similarly, proliferation of enthesiophytes and osteophytes along a joint surface would rightly be attributable to arthritis. There is a growing wealth of evidence that supports that pneumatic non-avian dinosaurs possessed avian-style respiration^55–61^. It follows then that since the pathologic areas in MOR 7029 are restricted entirely to the uniquely archosaurian pneumatic architecture, that this would be the focus of developing pulmonary differential diagnoses.

Within extant dinosaurs (Aves), pulmonary disorders are fairly common and come in a variety of forms^62^. Many of these diseases are diagnosed via soft tissue evaluation, and a few are known to affect underlying bone^37^. The lack of fresh soft tissue makes the exact diagnosis of the lesions in MOR 7029 quite challenging; however, we can narrow the list to three differentials – some with more evidentiary support for an etiologic agent than others.

### Neoplasia

#### Air sac carcinoma

Tumors of the avian respiratory tract are uncommon with fewer still reported within the air sacs. Occasional case reports exist in the literature with examples including air sac neoplasia of the sternum described in a quaker parrot^63^ and of the humerus air sac of a salmon-crested cockatoo^64^ and a Timneh African grey parrot^65^. These cases varied between no bone involvement to bone lysis and periosteal reaction. “Superficial spiculated bone” which may or may not represent periosteal bone proliferation was noted with no normal bone structure noted in one case^63^. Monostotic air sac neoplasia involvement has been described with some cases of pulmonary neoplasia metastasizing to ribs^66,67^, but multicentric air sac neoplasia has not been described in birds.

#### Pulmonary neoplasia

Adenocarcinoma of the pulmonary parenchyma (lung) has been occasionally described with a unique form of pulmonary pathology described in cockatoos^66–68^. In both cases, pulmonary neoplasm can locally invade the air sacs lying adjacent to the affected lung and/or thoracic vertebrae that are in direct contact with the lung^66–68^.

While macroscopic and CT based analyses have been conducted on MOR 7029, histopathology of the lesions has not been performed. The polyostotic distribution of changes limited to the vertebral pneumatic fossae in MOR 7029 do not fit with a primary neoplasm either within the vertebrae themselves or within the adjacent soft tissues or air sacs. Metastatic disease could be a consideration; however, in most avian cases, involvement of bone is due to either to a monostotic primary lesion, or when polyostotic is due to metastatic disease which is lytic rather than proliferative.

A final note on the possibility of fossil cancers: while tumors have been reported in the dinosaurian fossil record^33,34,47,54,69^, certain aspects of their life history may have curbed the proliferation of cellular degeneration. As cancer is abnormal cell growth, in theory, the more cells an organism has, the statistically higher probability that some portion will experience abnormalities. So, based on this principle, the largest organisms (which have the most cells), should have the highest rates of cancers. But this is not universally the case. Peto’s Paradox is a medical observation that cancer can inversely correlate with the number of cells, and that larger body size coupled with longevity may be able to ‘combat’ cancer^70^. Both elephants and whales are extremely large-bodied, long-lived mammals, yet cancers in these organisms are exceedingly rare, compared to its abundance in smaller, shorter life-spanned rodents^70^. In regard to the dinosaurian record, Tollis et al.^70^ noted that the occurrence of cancers in certain dinosaurs (1 out of 16 in *Edmontosaurus*) indicates its historical commonality. But while *Edmontosaurus* is a large-bodied vertebrate, in comparison to equally sized mammals, it had a proportionally shorter longevity^71^. Tollis et al.^70^ further speculated that given body size:age, certain dinosaurs (like *Edmontosaurus*) may not have evolved or replicated cancer-suppressing genes (as seen in long-lived organisms today). Since their lifespan was proportionally much shorter, these dinosaurs simply negated the need to evolve resistance to cancers. And given their increased body size and life history strategies, sauropods may have evolved some more rudimentary forms of cancer suppression (potentially supported by the significantly rarer occurrence of tumors in Sauropoda^70^).

### Airsacculitis

Airsacculitis is inflammation or infection of the air sacs, often due to actinobacterial, bacterial (such as *Escherichia coli* or *Mycoplasma*), fungal infections, or viruses. Airsacculitis may occur as a disease process on its own but is frequently diagnosed alongside pneumonia, bronchial disease and/or rhinitis and sinusitis. Several bacteria or fungi are known to cause airsacculitis^72–74^.

#### Mycobacteriosis

A disease commonly caused by the actinobacteria *Mycobacterium avium*, *M*. *fortuitum*, *M*. *genavense*, and *M*. *intracellulare*, it often causes the formation of granulomatous lesions in the liver and gastrointestinal tract^37,75–77^. While predominantly found in the liver and gastrointestinal tract, these lesions can less frequently occur in the spleen, kidney, pneumatic tissues, and bone marrow^37,76,77^. In these cases, (even in the marrow) these lesions do not ossify—instead the granulomas comprise of an eosinophilic matrix, multinucleated cells, macrophages, and heterophils^37,76,77^. In a very small percentage of mycobacteriosis cases (3.8%^75^), osseous growths consisting of osteomyelitis and osteophytosis were found along previous arthritic joints and fractures, and along the diaphysis of long-bones^37^.

While known to affect the same portions of the respiratory tract as MOR 7029, the non-osseous lesions and limb targeting bony pathology of mycobacteriosis are not consistent with the features expressed in MOR 7029. Therefore, we do not hypothesize that mycobacteriosis is the causal agent in MOR 7029.

However, Brum et al.^78^ reported an aeolosaurine titanosaur rib from the Upper Cretaceous of Brazil affected by what they identified as tuberculosis due to a *Mycobacterium* infection, which if a correct diagnosis, would make it the first confirmed case in a non-avian dinosaur. Consisting of a bony outgrowth on the medial surface of a dorsal rib, Brum et al.^78^ were able to histologically sample the element. This revealed abnormal tissue, associated with the external feature, that permeated endosteally into the otherwise ‘normal’ osseus microanatomy. Though Brum et al.^78^ noted that further discussion was needed to differentiate this structure in question from the possibility of pathologic medullary tissue (see citations within Brum et al.^78^), this current study ultimately concurs with the results of Brum et al.^78^ in that this dorsal rib shows evidence of a respiratory-related issue that was hypothetically bacterial in origin.

#### Chlamydiosis

Caused by the bacteria *Chlamydia*, chlamydiosis affects the respiratory and pneumatic tissue and causes flu-like conditions and pneumonia^37,79^. Though most commonly cause by *C*. *psittaci*, the other eight species, *C*. *abortus*, *C*. *caviae*, *C*. *felis*, *C*. *muridarum*, *C*. *pecorum*, *C*. *pneumoniae*, *C*. *suis*, and *C*. *trachomatis* can cause clinical chlamydiosis as well^67^. Interestingly, today at least, birds are a vector for chlamydiosis and it is a highly contagious zoonotic disease^80,81^. Generally, an accumulation of fibrin and inflammatory cell debris gather and thicken within the respiratory tract^37^. As in mycobacteriosis, the primary pathologic structures are not osseous, but they do occur throughout all of the respiratory tissue^37^.

#### Aspergillosis

Caused by the fungal genus *Aspergillus* (and often attributed to *A*. *fumigatus*, *A*. *flavus*, *A*. *niger*, *A*. *glaucus*, and *A*. *nidulans*), aspergillosis is the most common avian disorder that compromises the respiratory system and often causes granulomatous pneumonia^82^. Interestingly, from a paleontology perspective, the apparent first scientific account (though undiagnosed) of aspergillosis was by Richard Owen in 1832 when dissecting a flamingo^83^. Often causing yellowish-white to greyish-yellow non-osseous lesions throughout the respiratory system, similar to chlamydiosis, infections in the eyes, brain, skin, joints, and organs are also reported^82^. While rarer, aspergillosis in developing chicks has been documented to cause vertebral deformities^82^. *Aspergillus* is an incredibly common fungus, and particularly thrives in warm, humid, ‘swampy’ environments^82^. Though not zoonotically transmissible, aspergillosis is known in cats, dogs, horses, cows, dolphins, and humans^84^. Interestingly, in birds, health and life history factors are important, but without medical treatment, aspergillosis is lethal^83^.

As in chlamydiosis, aspergillosis effects and generally causes non-osseous lesions throughout the entire avian respiratory and pneumatic tract, although atrophic rhinitis can occur.

### Pneumoconiosis

Pneumoconiosis consists of the inhalation of dust or other airborne particulates which causes pulmonary fibrosis^85^. Common modern particulate causes of pneumoconiosis are silicosis and asbestosis^85^. At Ashfall Fossil Beds State Historical Park, continued inhalation of volcanic ash is hypothesized to have killed over 200 animals^86^. Many of the mid- to large-sized mammals at the site exhibit hypertrophic osteopathy (HO)^87^. These HO-associated proliferations—which formed on numerous cranial, axial, and appendicular elements—consist of spongy bone formations on top of normal cortical bone and localized bony inflammation; that are interpreted to have formed over the course of weeks to months^87^. Hypertrophic osteopathy is a recognized syndrome in humans, cats, dogs and horses and is frequently but not exclusively associated with intrathoracic pathology that may have neoplastic or infectious/inflammatory etiologies, or it may be occasionally seen associated with local disease involving vasculature that disrupts blood flow, such as arteriovenous fistulae. The exact pathophysiology is uncertain but reduced periosteal vascularity is frequently reported and it has been generally accepted that HO likely develops in part as a result of afferent neurologic stimulation^88,89^.

With regards to volcanism and the potential for pneumoconiosis, volcanic events are known and recorded within the Morrison Formation^90^. The bone bearing horizons at the MOR 7029 locality were sampled in 2015 to determine the age of deposition via zircon based radiometric dating. An in-depth analysis of these results are in development (by S. McMullen), but preliminary reporting herein found only detrital zircons, none of which were Late Jurassic in age.

The hypertrophic osteopathic proliferations present at Ashfall Beds occur on several elements not adjacent to nor a part of the respiratory tract (e.x. tibiae of *Teleoceras*; Bundy and Matson, 2013) and, given that no ‘active’ volcanic ash is present (i.e., non-detrital), nor is the site a *Konservat*-*Lagerstätte* (dozens to hundreds of animals with similar bony pathology are not present, like Ashfall Beds), we do not believe that pneumoconiosis was responsible for the bone pathology observed in MOR 7029.

#### Cause of pathology?

As stated before, we are highly unlikely to pinpoint the exact causation of the bony pathology in MOR 7029. Precise medical identification in the fossil record is incredibly rare. Identification can be especially problematic when a lesion lacks features consistent with a single disorder, or conversely, expresses features that are present in several conditions. The lack of precise identification means that several of these lesions can only tentatively be identified as “-like” (such as the “tuberculosis-like” bony pathologies reported in Surmik et al.^38^).

Additionally, while numerous bony pathologies exist that demonstrate the health struggles of a single individual (such as broken and healed bones), the odds of remains entering the fossil record indicates that such a disease should not have been exceedingly rare within a population to begin with (sensu^70^).

While pneumoconiosis and air sac carcinomas have some features similar to that of the MOR 7029 bony pathology, attributes inducing this condition (such as volcanic eruptions for pneumoconiosis), and especially prevalent secondary structures (localized osteomyelitis or bone/periosteal proliferations) are not consistent nor seen in MOR 7029. At this time, in light of the current evidence at hand, this analysis does not find pneumoconiosis, undifferentiated pulmonary tumors, nor air sac carcinomas as the most likely differential diagnosis for the pneumatic bony pathology in MOR 7029. We therefore, tentatively propose airsacculitis with associated osteomyelitis as the most parsimonious explanation. Admittedly, the lack of documented vertebral osseous pathologies with airsacculitis is potentially problematic, yet given the additional factors, our analysis finds it to be the most likely scenario for the interim.

Mycobacteriosis, chlamydiosis, and aspergillosis are extremely common and virulent today. And, opposed to explanation due to a virus or paleoepizootic, these diseases originate from mycobacteria and fungi already occurring within the natural environment. Given the susceptibility and prevalence of chlamydiosis and aspergillosis (the most common respiratory disorders in birds), one cannot but suspect a similar causation agent in another saurischian dinosaur. This is especially true for aspergillosis, considering its prevalence and the environment it occurs in. While portions of the Morrison Formation were characterized by seasonal aridity^90^, this was not the case of the northern extent. The northern extent was closer in proximity to the retreating Sundance Sea, and this more coastal region was warm and humid^39,40,90–92^—environmental conditions today perfect for the *Aspergillus* fungi. And while not Mesozoic in age, there are Eocene fossil *Aspergillus* spores reported from Baltic amber^93^.

It is tempting to ascribe the pneumatic bone pathology of MOR 7029 to aspergillosis causing pneumonia; doing so would establish a fossil record for this disease and highlight yet another shared bird/dinosaur disease. However, as mentioned throughout this analysis, the lack of tissues/structures that elucidate origination means that we cannot pinpoint a causation at this time. Therefore, our suggestion of airsacculitis with associated osteomyelitis—a respiratory disorder with secondary osseous inflammations, likely from an infection—is the most conservative answer.

However, even with a conservative diagnosis of airsacculitis, we can hypothesize the symptoms the animal would express. Both chlamydiosis and aspergillosis cause respiratory disease in birds today, and the symptoms include weight loss, coughing, nasal discharge, fever, labored breathing, diarrhea, lethargy and death^37,82,94^. MOR 7029 could have expressed these same symptoms, and given these physical tolls, it is a plausible scenario that the animal ultimately succumbed to and died from this respiratory infection.

Supporting this interpretation is the possible *Mycobacterium* induced tuberculosis-like disease reported by Brum et al.^78^. Brum et al.^78^ likewise inferred that their titanosaur suffered from the symptoms of pneumonia—though it was not theorized if this led to the animal’s demise. Furthermore, Brum et al.^78^ noted that internally, this reactive tissue also pervaded into the pneumatic structures. Titanosaurs are known to have had extensive postcranial pneumatization^95,96^ that likewise incorporated the dorsal ribs into the pulmonary system. Both the cases presented by Brum et al.^78^ and herein demonstrate irregular, osseus proliferation with internal reactive tissues that are associated with pneumatic structures. Given that the aeolosaurine rib of Brum et al.^78^ possessed a pneumatic foramen and complex internal pneumatization, we would further state that the external bone proliferation and reactive tissues etiologically supports an avian-like causal mechanism for infection in this sauropod as well.

#### Implications for the sauropod respiratory system

In the previous section, we attempted to diagnose the cause of the lesions in MOR 7029—to ask what the pneumatic-associated morphology of this sauropod might tell us about the disease process. We can also ask the converse: do the lesions in MOR 7029 contribute to our knowledge of the sauropod respiratory system?

**Figure 3:**
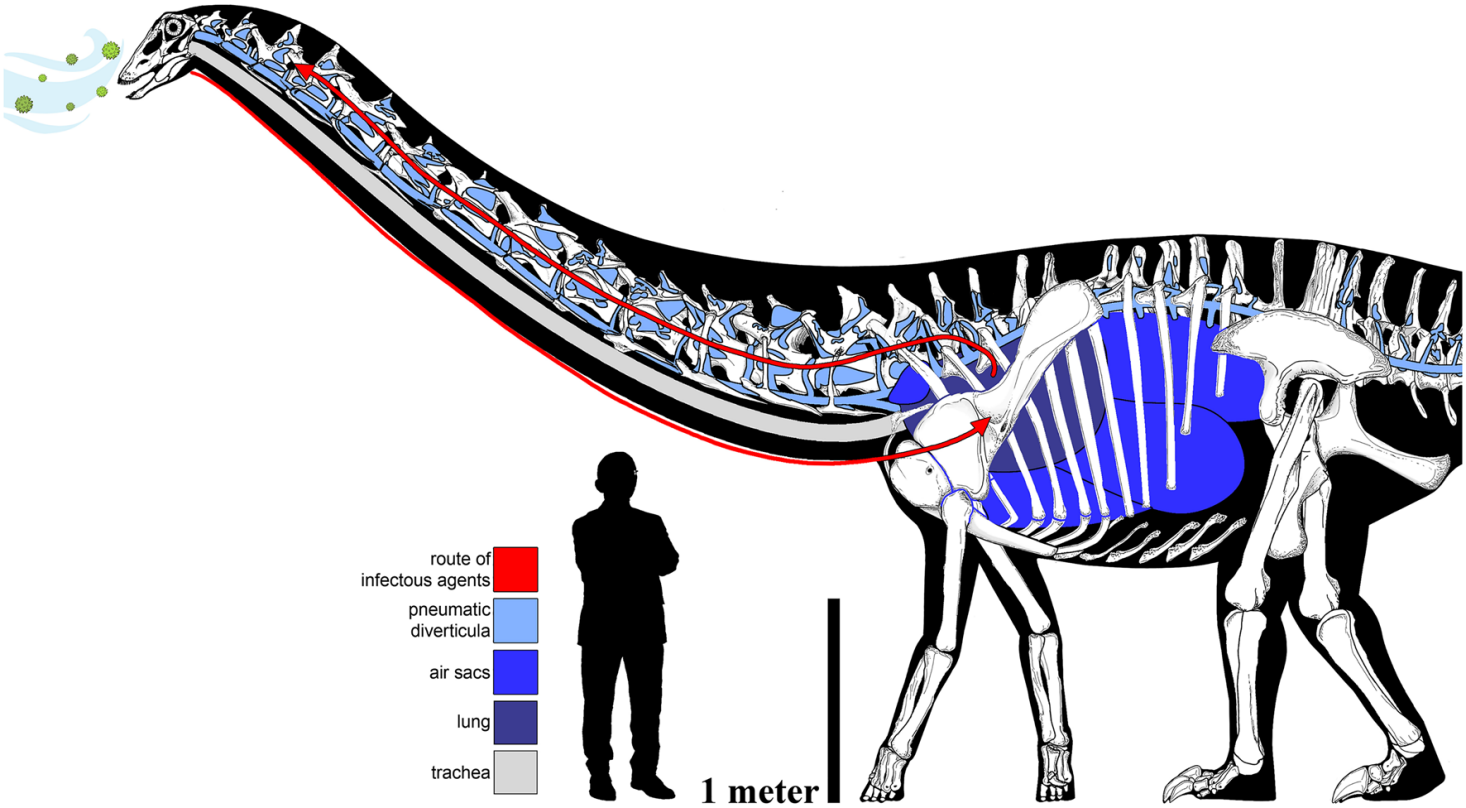
The elaborate and circuitous pulmonary complex of the sauropod, with the hypothetical route of infectious pathway in MOR 7029. Skeletal reconstruction of the diplodocine *Galeamopus pabsti* by and copyright of Francisco Bruñén Alfaro to scale with MOR 7029. Human scale bar is the exemplar of pandemic education and rationalism, Dr. Anthony Fauci, at his natural height of 170 cm.

A starting point is to recognize that in diplodocine sauropods, much of the vertebral column was pneumatized, including all of the post-atlantal cervical vertebrae, all of the dorsal vertebrae, most or all of the sacral vertebrae, and, in *Barosaurus* and *Diplodocus*, approximately the first 19 caudal vertebrae^42,97^. Given what we know from pneumatic bones in extant amniotes, it is reasonable to assume that all of those vertebrae needed a patent connection to the outside air, via the pneumatic diverticula, respiratory air sacs, lungs, and trachea, for their pneumatic spaces to develop and be maintained^98^. So, the anatomical domain that was potentially vulnerable to airborne pathogens or respiratory illness in these sauropods was vast, including almost all of the neck and trunk and much of the tail (Fig. 3).

In that context, three points about the lesions in MOR 7029 deserve attention: (1) the lesions occur in the cranial half of the neck, relatively distal to the lungs and respiratory air sacs in the trunk; (2) the lesions occur in several serially-adjacent vertebrae; and (3) the lesions occur bilaterally within each affected vertebra.

##### Distance from the thorax

Although hematogenous (blood-borne) pneumonia exists in both humans and animals, we think it is a far less likely explanation for the lesions in MOR 7029. Hematogenous pneumonias commonly occur bilaterally in the lungs, which is consistent with the bilateral distribution of the lesions in this specimen; but it is difficult to explain why blood-borne rather than airborne agents would have settled bilaterally in such a localized area, so far from the lungs and air sacs. The network of pneumatic diverticula around the cervical vertebrae in diplodocines was probably extensive^99^, and that network created a potential highway for the spread of airborne pathogens far beyond the core respiratory system. Similarly, extensive diverticular networks exist in the vertebral columns of many extant birds^100^; although, we know very little about the rate at which air is exchanged in pneumatic diverticula that are distant from the lungs and air sacs. Even if the diverticular network was extensive, it may have still been an anatomical ‘cul-de-sac’, with little or no active circulation of air, and this may have made the vertebral diverticula particularly susceptible to infection.

##### Serially-adjacent vertebrae

In extant birds, the cervical vertebrae are pneumatized by diverticula of the cervical air sacs that develop anteriorly from the thorax and pass through the transverse foramina (the bony loops formed by the fused cervical ribs) along with the vertebral arteries. These bilaterally paired diverticula have been referred to as diverticula intertransversaria^101^ and lateral vertebral diverticula^100^, and they are inferred to have been present in sauropod and non-avian theropod dinosaurs. Because the lateral vertebral diverticula ran adjacent to all of the pneumatic cervical vertebrae, they could have created a sort of ‘infection superhighway’ for pathogens not only to reach the middle of the neck, but also to spread among adjacent vertebrae. That the lesions in MOR 7029 are all in the lateral-facing pneumatic cavities of the centra, which directly faced the lateral vertebral diverticula, suggests that the lateral vertebral diverticula themselves were infected. The vertebral lesions are the osteological footprints of a possibly more extensive respiratory illness; in other words, Cv 5–7 were not the only vertebrae that were infected, rather they were the only vertebrae in which the infection was severe enough to produce an osteological reaction.

##### Bilateral lesions

**Figure 4:**
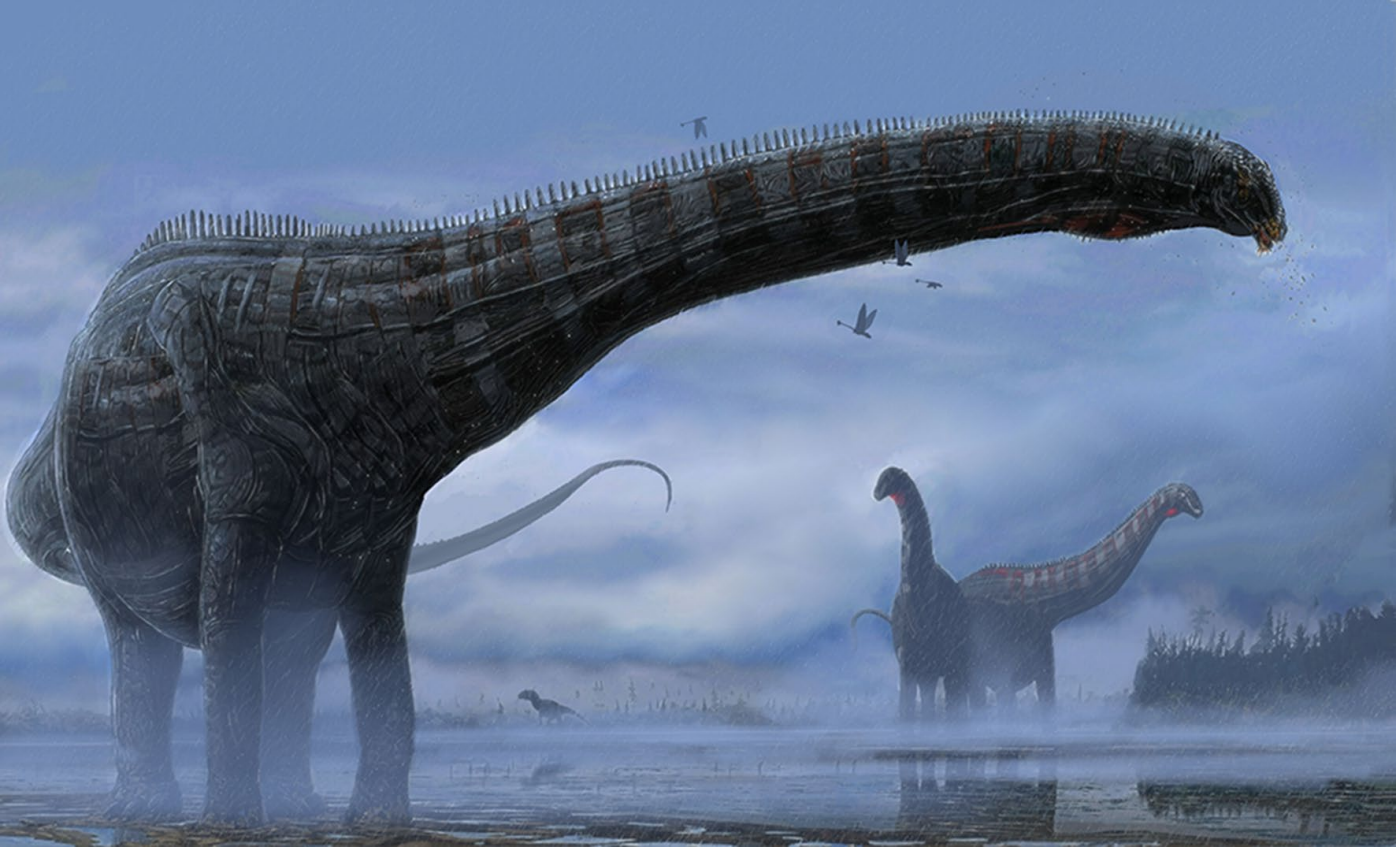
Hypothetical life restoration of MOR 7029. Note that the pulmonary disease infecting this animal would not been externally evident, but the probable pneumonia-like outward symptoms would have included coughing, labored breathing, nasal discharge, fever, and weight loss among others. Artwork by and copyright of Corbin Rainbolt. Image may not be used for any commercial purposes.

If the cervical vertebrae of sauropods were pneumatized by diverticula passing anteriorly through the transverse foramina, as occurs in birds, then the left and right sides of each vertebra were pneumatized independently, at least in the initial stages. Zurriaguz and Álvarez^102^ found consistent asymmetry of pneumatic features along the cervical series in the titanosaurian sauropods *Neuquensaurus* and *Saltasaurus*, which is circumstantial evidence for independent pneumatization on the left and right sides of the vertebral column. In extant birds, the lateral vertebral diverticula on either side of the neck can become connected over the course of ontogeny by anastomotic diverticula that penetrate medially to contact the spinal cord^100,101^. The clustering of pneumatic features around the neural canal in sauropods, non-avian theropods, and pterosaurs (Ref.^103^: Fig. 4) suggests that similar diverticula were present in those lineages. The presence of presumably infectious lesions bilaterally in Cv 5–7 of MOR 7029 is another line of evidence that the pneumatic diverticula on the left and right sides of the neck had an anastomotic communication. In the absence of such a communication across the animal’s midline, it would be an extreme coincidence that in such a long neck, at such a great distance from the thorax, exactly the same vertebrae would be infected bilaterally.

Additionally, another piece of supportive evidence for penetrating and anastomosing diverticula are the many sauropod specimens that exhibit additional vertebral openings. *Camarasaurus supremus* (AMNH 5761/X1), *Diplodocus carnegii* (CM 84), *Galeamopus pabsti* (SMA 0011), and *Smitanosaurus agilis* (USNM 5384) are among the many specimens which exhibit pierced medial septa with smoothed and rounded openings, while *Sauroposeidon proteles* (OMNH 53062) exhibits a similar feature in fossae on the lateral side of the neural arch. But do such perforations dictate pneumatic penetration? Openings in bones are not uncommon, and while being covered by the periosteum, such features would constitute fenestrae, not foramina. However, perhaps, pneumatic diverticula were able to remodel the medially adjacent bone and were able to laterally ‘bridge’ the septum, opposed to representing an opening in which the diverticula passed through or bisected the vertebra.

Pneumatization of sauropod cervical vertebrae by bilaterally-paired vertebral diverticula, which were connected to the lungs and intra-thoracic air sacs, and connected across the midline by diverticular anastomoses, has previously been hypothesized by a number of authors. The lesions in MOR 7029 provide new evidence that the inferred network of soft-tissue diverticula was in fact present (Fig. 3).

## Conclusion

MOR 7029 documents the first case of an avian-like airsacculitis in a non-avian dinosaur. Osteologically expressed by proliferative bone projecting from the pneumatic fossae in anterior cervical vertebrae, this is the first lesion of its kind reported in a non-avian dinosaur. In comparison to extant medical cases, a majority of respiratory related diseases cause lesions in soft tissues (lungs, organs, etc.); therefore, comparison to fossil cases is difficult. However, the location and restriction of the pathologic structures in MOR 7029 strongly indicate a respiratory-related issue.

In comparison to modern birds, there are several biotic and abiotic factors that can compromise the respiratory system. Many of these disorders can result in bone proliferation (as in MOR 7029), but the causal mechanism does not match (such as pneumoconiosis from regional volcanism). In a review of avian respiratory disorders, at this time we find airsacculitis to be the most parsimonious explanation. Several causations of airsacculitis are natural agents and occur today with prolific frequency (chlamydiosis and aspergillosis). While several circumstantial factors could be interpreted to have come from a specific agent—such as aspergillosis—at this time it is more conservation to diagnose MOR 7029 as airsacculitis with associated osteomyelitis.

Even with a tentative diagnosis of airsacculitis, this constitutes the first identification of this disease in a non-avian dinosaur. Given that airsacculitis-causing disorders (like aspergillosis) can occur in both reptiles and birds, this diagnosis is phylogenetically supported. In consideration of the prevalence at which it occurs today, and being a naturally stemming disease, we can now consider airsacculitis as being a disorder in dinosaurs. At this time, we do not have evidence of air sacculitis in basal dinosauriforms and archosauriforms, therefore, we cannot speculate how early airsacculitis evolved and began to affect archosaurs. However, a Late Jurassic diagnosis establishes the first fossil record of this disorder. With more and continuing investigations, we will hopefully be able to (1) continue to document and trace medical conditions present today, (2) better our understanding of the disorders that affected the dinosaurs, and (3) understand how the identification of these disorders contribute to our understanding of dinosaurian physiology (Fig. 4).
